# Impact of Glucose on the Nanostructure and Mechanical Properties of Calcium-Alginate Hydrogels

**DOI:** 10.3390/gels8020071

**Published:** 2022-01-22

**Authors:** Patricia Lopez-Sanchez, Ali Assifaoui, Fabrice Cousin, Josefine Moser, Mauricio R. Bonilla, Anna Ström

**Affiliations:** 1 Department of Chemistry and Chemical Engineering, Chalmers University of Technology, 412 96 Gothenburg, Sweden; anna.strom@chalmers.se; 2 Unité Mixte de Recherche Procédés Alimentaires et Microbiologiques, Université de Bourgogne Franche-Comté (UBFC), UMR PAM A 02.102, 21000 Dijon, France; Ali.Assifaoui@u-bourgogne.fr; 3 Laboratoire Léon Brillouin, Université Paris-Saclay, UMR 12, CEA-CNRS, 91191 Gif Sur Yvette, France; fabrice.cousin@cea.fr; 4 Department Bioeconomy and Health, Research Institutes of Sweden RISE, 412 76 Gothenburg, Sweden; josefinemoser@gmx.de; 5 Basque Center for Applied Mathematics BCAM, 48009 Bilbao, Spain; mrincon@bcamath.org

**Keywords:** alginate, glucose, hydrogels, SANS, crosslinking, poroelasticity, mechanical properties, structure

## Abstract

Alginate is a polysaccharide obtained from brown seaweed that is widely used in food, pharmaceutical, and biotechnological applications due to its versatility as a viscosifier and gelling agent. Here, we investigated the influence of the addition of glucose on the structure and mechanical properties of alginate solutions and calcium-alginate hydrogels produced by internal gelation through crosslinking with Ca^2+^. Using ^1^H low-field nuclear magnetic resonance (NMR) and small angle neutron scattering (SANS), we showed that alginate solutions at 1 wt % present structural heterogeneities at local scale whose size increases with glucose concentration (15–45 wt %). Remarkably, the molecular conformation of alginate in the gels obtained from internal gelation by Ca^2+^ crosslinking is similar to that found in solution. The mechanical properties of the gels evidence an increase in gel strength and elasticity upon the addition of glucose. The fitting of mechanical properties to a poroelastic model shows that structural changes within solutions prior to gelation and the increase in solvent viscosity contribute to the gel strength. The nanostructure of the gels (at local scale, i.e., up to few hundreds of Å) remains unaltered by the presence of glucose up to 30 wt %. At 45 wt %, the permeability obtained by the poroelastic model decreases, and the Young’s modulus increases. We suggest that macro (rather than micro) structural changes lead to this behavior due to the creation of a network of denser zones of chains at 45 wt % glucose. Our study paves the way for the design of calcium-alginate hydrogels with controlled structure for food and pharmaceutical applications in which interactions with glucose are of relevance.

## 1. Introduction

Alginate is found in the extracellular matrix of brown algae, and it has been used for decades as a thickener, gelling agent and encapsulation material [1] for food [2,3,4], pharmaceutical [5,6] and biotechnological applications [7,8].

Alginate is a charged and linear copolymer consisting of (1–4) linked β-D-mannuronic acid (M) and α-L-guluronic acid (G), whose ratio varies depending on the alginate source. Due to its charged nature, alginate chains can form gels in the presence of low concentrations of di- and trivalent cations (Mg^2+^ being an exception) at a range of pH values and temperatures. The gelation properties of the alginate depend on the M/G ratio as well as the distribution of the M and G units. Calcium-mediated gelation has been broadly studied and is attributed to the chelation of Ca^2+^ between G units from different alginate chains via the so-called egg-box model [9]. The egg-box model involves a two-step network formation mechanism, a dimerization process followed by dimer–dimer aggregation of G units and Ca^2+^ [10]. The egg-box model has been revisited since its introduction, with the proposition of a mono-complexation between alginate and Ca^2+^ [11] or multicomplex modality [12] prior to dimerization and dimer–dimer aggregation. The ion-induced gelation ability of alginate is determined by the total amount of G units and its distribution along the chain, where the high amount of G units and GG blocks are favorable [13,14]. The microstructure of the calcium-alginate gels depends on the type of gelation process and determines its physicochemical properties and functionality. Two routes of crosslinking the chains can be envisaged. The so-called external gelation [13] approach is an addition of alginate to a CaCl_2_ solution bath, in which Ca^2+^ diffuses to the interior of the alginate solution, forming a gelled alginate matrix from the outside to the center. A second approach is the internal gelation, in which an insoluble calcium salt, such as CaCO_3,_ is added to the alginate solution in the presence of glucono-δ-lactone (GDL), which acidifies the solution and releases Ca^2+^ from the insoluble salt for crosslinking with the alginate. Gels produced by internal gelation have been shown to be more homogeneous than by external gelation, because it prevents the formation of gradients concentration that occur with the external approach. In general, it has been shown that for a fixed amount of alginate, an increasing amount of Ca^2+^ leads to an increased gel modulus [15], while the fracture strain is independent of both alginate and calcium concentration [16].

Studies on the effect of sugars, or other co-solutes, on the gelation of polysaccharides are of importance for the food industry, as there is a demand to replace the sugars, fat, and salt in foods. Sugars play a role in food taste but also in food structure [17]; therefore, the reduction of sugar can be technically challenging. The role of sugars in sport nutrition and its optimal delivery to the body is another aspect where the impact of sugars on polysaccharides conformation is of importance [18,19,20].

When sugars are dissolved in an aqueous polysaccharide solution, they restructure the water molecules, increasing the local concentration of polysaccharides. Moreover, the presence of sugars and other polyols [21] may also induce changes in polysaccharide molecular conformations [22], as they can bind water in their hydration shells and reduce the amount of water surrounding the polysaccharides. The nature of the sugar molecules, which is related to the number of equatorial OH groups, is key to determining their impact on the development of the network structure [17]. The interactions of sugars via hydrogen bonding are more probable with flexible molecules compared to stiff rods for entropic reasons, and they can also reduce the hydration state of the polysaccharides, which is required for the thermodynamic stability. For agarose, a common texturizer used in foods, it has been shown that the stiffness of agarose gels increases with the addition of up to 40 wt % sugar (sucrose and glucose) due to the stabilizing effect of the sugars on the agarose chain associations; whilst at higher concentration of 80 wt %, a phase inversion occurred, in which the agarose network converted to a continuous sugar phase with agarose present as dispersed inclusions, drastically decreasing the gel storage modulus [23,24]. Agarose gels produced in the presence of up to 40 wt % fructose or sucrose showed an increase in elastic modulus; however, at 60 wt % fructose, the elastic modulus of agarose gels increased, whilst the same level of sucrose drastically decreased the elastic modulus [25]. Along the same lines, the addition of sugars increased elastic modulus, which has been shown for gellan and kappa-carrageenan at sucrose concentrations up to 30 wt % [26,27]. In calcium-alginate-chitosan gel particles, strain at failure of gels containing 30 wt % sucrose was higher than for lower sucrose concentrations [28]. These studies highlighted that the complex underlying molecular processes behind polysaccharide gelation in the presence of these co-solutes (i.e., solvent quality effects, competition for water, and direct/physical interaction between sugar and polysaccharide) depend on the sugar type and concentration.

Previously, we investigated the impact of sugar (glucose:fructose mixtures) as co-solutes (15–60 wt %) on the physicochemical properties of calcium-alginate spherical gel particles produced by external gelation [19]. Our findings showed that sugar concentrations of 30–60 wt % reduced the intrinsic viscosity of the alginate polymer in dilute solution, and the gel network appeared more open and less connected. In order to better understand the impact that low molecular weight sugars have on the structure and gelation properties of calcium-alginate gels here, we further investigate the influence of glucose as a co-solute on the nano and microstructure of calcium-alginate gels produced by internal gelation. In our approach, we first explored the structural features of alginate in glucose solutions of increasing concentrations up to 45 wt % and in calcium-alginate gels using ^1^H-low field nuclear magnetic resonance (NMR) and small angle neutron scattering (SANS); subsequently we investigated the gel mechanical properties using compression and creep tests and fitted them to a poroelastic mechanical model. Exploring the relationship between structure and gel properties provides a better understanding of the mechanism of alginate gelation in the presence of glucose and aids the design of biopolymer gels for food industry and pharma applications.

## 2. Results and Discussion

### 2.1. Intrinsic Viscosity of Alginate

The intrinsic viscosity [η] for alginate in 0.1 M NaCl was measured to be 0.29 ± 0.01 mL/mg (Equations (1)–(4) and Figure S1). This value is lower than previously reported for other types of alginate with higher G content performed in our lab of 0.54 mL/mg [21], 0.9 mL/mg [19], and lower than a large range of alginate with reported values of [η] of 0.52–1.44 mL/mg at 0.1 M NaCl and T = 20 °C [15]. The molecular weight of the alginate can be calculated from the Mark–Houwink equation, [η] = KM^a^. Using K = 0.0051 and a = 1.00 [29], a molecular weight of 57 kDa is obtained for the alginate used here.

### 2.2. Effect of Glucose on the Water Activity and Proton Mobility in Solutions and Gels

**Figure 1:**
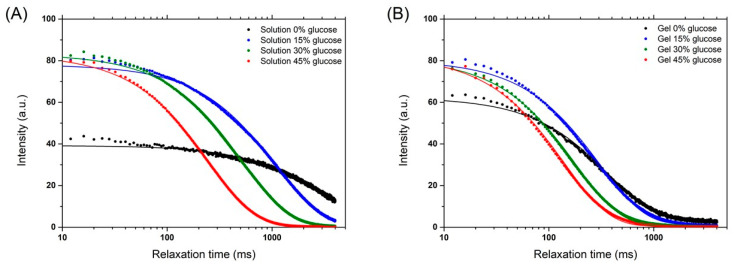
NMR CPMG signal for alginate solution (**A**) and calcium-alginate hydrogels (**B**) with different glucose concentrations (0, 15, 30, and 45 wt %). Lines represent the fit of experimental data by using one single exponential, as shown in Equation (5). Notice that the intensity at low relaxation times is the same for all glucose samples due to saturation of the signal.

**Figure 2:**
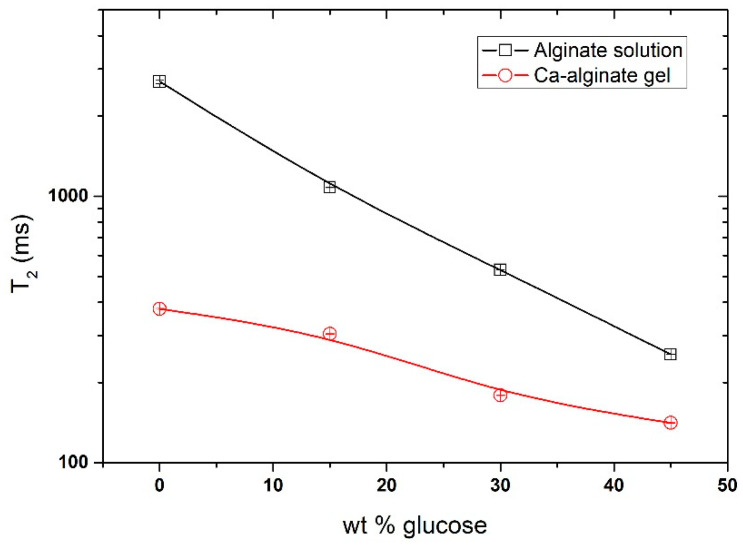
Spin–spin relaxation time for alginate solution and calcium-alginate gel as a function of glucose concentration (0, 15, 30, and 45 wt %).

Fourier Transform Infrared (FTIR) showed various peaks when glucose was added to the alginate solution, which could be assigned to the different vibration bands of glucose (Figure S2). The intensity of such peaks increased proportionally with the increase in glucose content, indicating that the added glucose is equally distributed within the alginate solution, both in solution and once the gel is formed. Furthermore, as expected, the addition of glucose to alginate solution has a strong effect on the water activity (a_w_), which decreased proportionally with the increase in glucose content (Figure S3). The proton mobility in alginate solution and gels prepared in D_2_O with various concentrations of deuterated glucose was determined by ^1^H low field NMR (Figure 1). The intensity follows an exponential function where the intensity is related to the number of protons; then, the intensity decay was fitted by one exponential function (Figure 2), and the spin–spin relaxation time (T2) was extracted. For the 1 wt % alginate solution, without glucose, the number of protons is low, since the solution was prepared in D_2_O, and the spin–spin relaxation time (T2) was close to the T2 of water (T2 ≈ 2.5 s) (Figure 2). For both alginate and gels, it was observed that the increase in glucose concentration induced a decrease in T2, indicating that the presence of glucose reduces the mobility of protons. This reduction is more pronounced in calcium-alginate gels than in the solutions. The protons probed in this experiment are mainly those related to the alginate chains. The in situ release of calcium through the solubilization of CaCO_3_ by GDL induces the formation of a calcium-alginate network where the alginate chains are less mobile.

### 2.3. Determination of the Local Structure of Solutions and Gels by SANS

**Figure 3:**
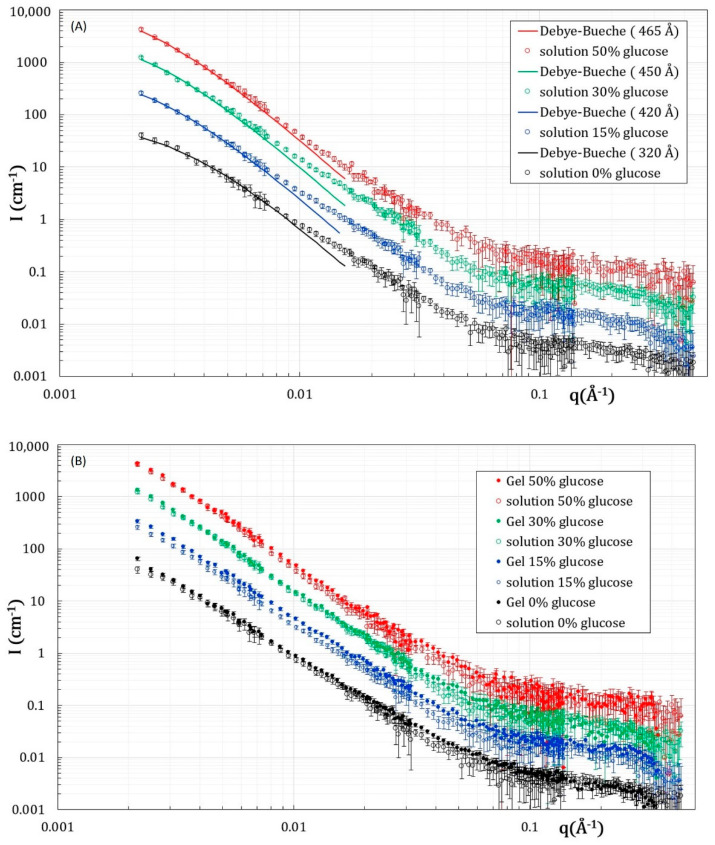
SANS scattering curves for solutions (**A**) and gels (**B**) prepared in D_2_O with deuterated glucose. The data for solutions and gels at 0% glucose are in absolute scale. The other scattering curve are shifted from one to another by a factor of 3.33. The continuous line in (**A**) is a fit of the low q part of the scattering by a Debye–Bueche approach. Errors bars are calculated from standard deviation.

The SANS scattering curves for solutions and gels at 1 wt % have been measured for 0, 15, 30, and 45 wt % deuterated glucose in D_2_O solvent. The use of deuterated glucose, almost contrast matched in D_2_O, ensures that the scattering comes only from alginate chains, as demonstrated by the scattering curves of solutions (Figure 3) that all overlap at large q, in the domain where only the form factor of chains is probed.

The scattering curves of all the solutions show the same behavior, with three clear different features as a function of the q-range: (i) at large q, I(q) decays similarly to q^−1^, which is typical from the scattering of a 1D object at such scale, due to the stiffness of the chain. (ii) At intermediate q, I(q) decays similarly to q^−3^ for all samples showing that there are large heterogeneities/fluctuations within the solutions. (iii) At the lowest q, the scattering tends progressively to a plateau, even if it is not reached in the probed q range, indicating that the fluctuations have a finite size.

The cut-off between the q^−1^ and q^−3^ occurs at the same *q*_co_ for all samples (Figure S4), which enables determining the persistence length of the chains. Thus, there is no change of the local rigidity of the chains in the presence of various amounts of glucose. However, it should be noted that the persistent length *L*_p_ is here apparent, with *L*_p_~6/(π *q*_co_), since the chains are not in a diluted regime. The value of *q*_co_ is ≈0.065 Å^−1^, which gives an *L*_p_ of 29.3 Å.

At the low q, the onset at which the q^−3^ decay tends toward a plateau, varies from one sample to another and is progressively shifted toward the low q with the increase in the glucose content.

In order to get an estimate of the characteristic size of such fluctuations/inhomogeneities, we successfully fitted the low q part of the scattered intensity to a Debye–Bueche function (Equation (6)). This model fits very well the experimental data with a characteristic size of inhomogeneities *Ξ* increasing from 320Å ± 20 Å, when there is no glucose, up to 420 Å ± 20 Å at 15 wt % glucose and 450 ± 20 Å at 30 wt % glucose. The introduction of glucose induces the formation of larger fluctuations. Above 30 wt % glucose, the changes induced by the further addition of glucose are small, since *Ξ* is 465 ± 20 Å at 45 wt %.

The structure of the gel without glucose is very similar to that of the solution used to make the gel, as their respective scatterings are very close (Figure 3B), except in the 0.02 Å^−1^ and 0.1 Å^−1^ regions, where a slight excess scattering is observed in the gel. In addition, at the lowest probed q, the scattering no longer tends to a plateau in the case of the gel (Figure 3B). Thus, the gel is more heterogeneous at large scales than the solution, and it is here impossible to obtain a size for the fluctuations by a Debye–Bueche approach, since such a size is out of the probed q-window. The excess of scattering observed at intermediate q is reminiscent of the shoulder that is classically observed by SANS in gels that display a given correlation size corresponding to the mesh. The observation of such a shoulder here demonstrates that a network is formed. The fitting of such a shoulder by a Lorentzian function (Ornstein–Zernike) usually enables obtaining the mesh size of the gels [30]. However, in the present case, any fitting would be senseless, given that the scattering is dominated by the large fluctuations existing in the system at larger scale.

**Figure 4:**
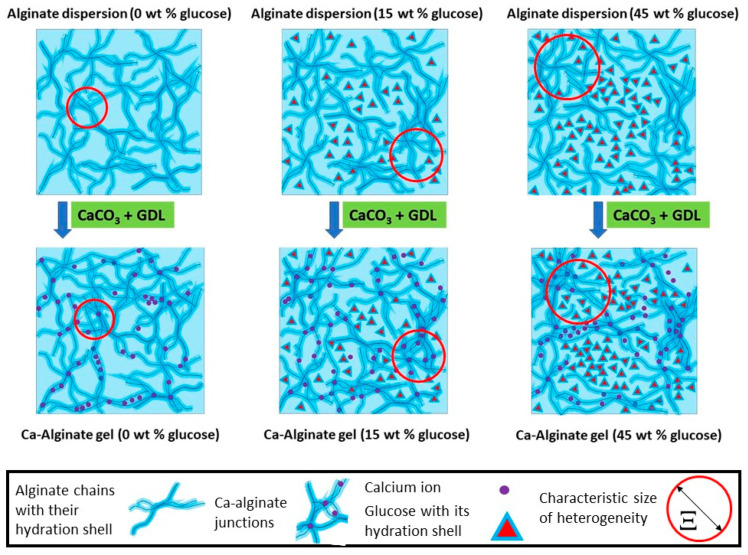
Schematic representation of the structure of the alginate solutions and calcium-alginate gels with different glucose concentrations.

In presence of glucose, the structure of gels is very similar to that of solutions at the same glucose content. For the 15 wt % glucose, there is only a very slight increase in the scattered intensity in the gel with respect to those of the solution. At larger glucose content (30% and 45 wt %), the scattering curves of the gel and solution overlap perfectly. Thus, the gelation of the alginate chains by Ca^2+^ cations has not induced any reorganization at the local scale, i.e., at lengthscales < 465 Å, which is the largest correlation length obtained in the case of 45 wt % glucose. Thus, the introduction of glucose modifies the microstructure of the solution prior to gelation at the local lengthscale. Figure 4 is a schematic representation of the proposed structure for the solutions and gels based on the results obtained from SANS measurements.

### 2.4. Mechanical Properties of the Gels and Poroelastic Model

**Figure 5:**
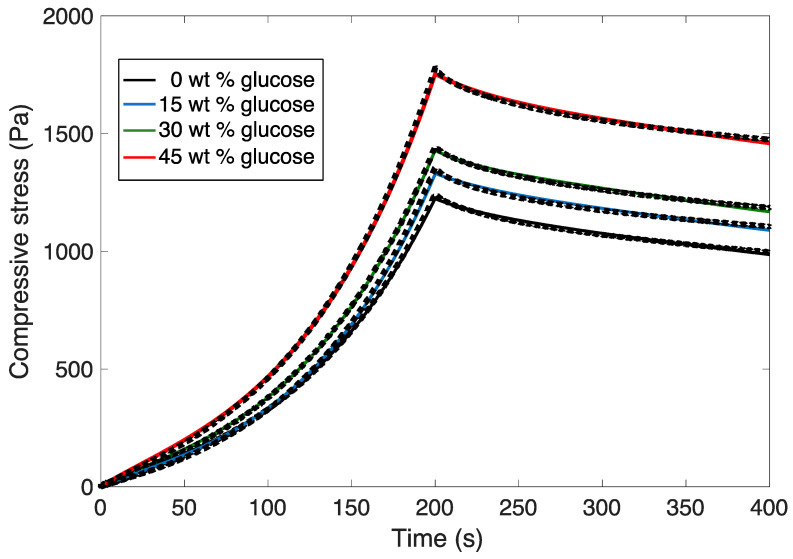
Compression–relaxation curves of calcium-alginate gels with different glucose concentrations. The dashed back line represents the experimental data. Solid lines are the model predictions.

Compression–relaxation curves are shown in Figure 5. An increase in glucose concentration increased the gel strength, which is represented by a higher maximum compressive stress. During compression, both structural deformation and the dynamics of fluid flow contribute to the mechanical behavior. The increase in viscosity of the fluid, as the glucose concentration increases, leads to a decrease in the ability of fluid to flow during compression and in turn to a higher pressure generated by the fluid, which thus contributes to an increase in the normal stress. In order to discriminate between structural and fluid dynamics effects, the compression–relaxation curves were fitted to a nonlinear poroelasticity model [31], which has been previously used to model the micromechanical behavior of alginate calcium gels [32]. Fitting curves are shown in Figure 5. We found that a Poisson’s ratio υ = 0.24 and γ = 0.65 provided perfect curve fitting for all samples. A Poisson’s ratio of υ = 0.31 has been previously reported for alginate-calcium gel beads [32].

**Figure 6:**
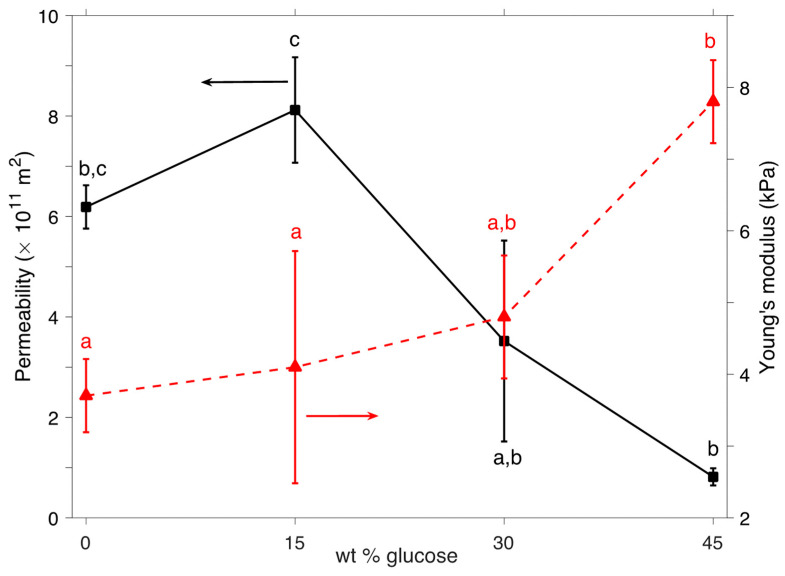
Young’s modulus, *E* (red triangles), and effective permeability, *k*/(*ϕ ^f^*)^2^ (black squares) extracted from the compression–relaxation experiments through the model in Equations (7) to (11). Error bars represent the standard deviation of three replicate measurements. Different letters represent significant differences (*p* < 0.05).

The variation in Young’s modulus E and effective permeability k/(ϕ f)^2^ with glucose concentration is shown in Figure 6. According to the model, the Young’s modulus remains essentially constant at 15 wt % glucose content, and it significantly increases at 45 wt %. The increase in Youngs moduli is not only due to viscous effects, which are accounted for independently in the model, but to possible (macro)structural changes within the samples. It is possible that at high glucose concentration, isolated pockets of alginate microgel structures form a spanning cluster, leading to sample strengthening without necessarily altering the local microstructure of the gels in a significant way. Figure 6 also shows the variation of permeability with glucose concentration. Here, permeability is related to pore size and tortuosity. There is a large variability between the replicates; however, the trend indicates a decrease in permeability with glucose concentration. A decrease in permeability as the viscosity of the fluid phase increases has been previously shown for cellulose gels filled with pectin solutions [33], indicating that the fluid phase controlled the permeability of those gels. Replacing the interstitial space between isolated microgel pockets by a spanning gel network may lead to the mild decrease in permeability observed here. If there had been a fundamental change in the gel microstructure (e.g., by decreasing the average pore size or the mean porosity through more intense crosslinking), such a decrease would expectedly be more dramatic. In addition, we note that the same values of the uniform nonlinear constant and Poisson’s ratio provide excellent curve fitting for all gels, supporting the argument that at sufficiently high glucose contents, although the isolated alginate networks tend to aggregate, the microgel structure is preserved.

## 3. Conclusions

Our results show that the apparent persistence length and the characteristic size of the alginate solution, obtained from SANS measurements and using the Debye–Bueche model, increase with glucose concentration, indicating increased heterogeneities on the lengthscale of hundreds of Ångström in the solutions. Interestingly, the scattering curves of the solutions and gelled networks are similar, suggesting that the features with a lengthscale of Å remain similar upon gelation. The fitting of the micromechanical measurements to a nonlinear poroelastic model revealed that the bulk microstructure of the gels is not significantly modified by the presence of glucose up to 30 wt %. However, at 45 wt % glucose, the permeability decreases, and the Young’s modulus increases, while the Poisson’s ratio and nonlinearity parameter γ remain constant. We propose that macro (rather than micro) structural changes lead to this behavior, and the effect of glucose on alginate solutions and gels is related to the solvent quality/water competition rather than direct interaction between glucose and alginate chains.

## 4. Materials and Methods

### 4.1. Materials

Alginic acid, D-(+)-glucose, sodium chloride, ethylenediaminetetraacetic acid (EDTA), and deuterium oxide (heavy water) were purchased from Sigma-Aldrich (St Louis, MI, USA); calcium carbonate was from Acros organics (Fisher Scientific, Waltham, MA, USA), and D-(+)-Gluconic acid-lactone was from Sigma Life Science (St Louis, MI,, USA). Deuterated D-glucose (1,2,3,4,5,6,6-D7, 97–98%) was purchased from Cambridge Isotope Laboratories Inc (Tewksbury, MA, USA). All chemicals had a purity of 98–99%.

Prior to use, alginate 0.5 wt % was dialyzed (Spectra/Por 7 membrane, Mw with a cut-off size of 15 kDa, Spectrum Labs, San Francisco, CA, USA) against deionized water (2 days) and against 0.01 M EDTA (2 days). After dialysis, alginate was freeze dried and stored at 4 °C for further experiments.

### 4.2. Intrinsic Viscosity

Alginate was added to 0.1 M NaCl to reach a concentration of 1 mg/mL alginate and dissolved at room temperature. From the 1 mg/mL alginate dilution, a series were prepared (0.2, 0.4, 0.6, and 0.8 mg/mL). The intrinsic viscosity was determined with an automated Ubbelohde viscometer (Schott-Geräte, Germany) with a 53101-0a capillary (SI Analytics, Germany). The measurements were performed at T = 25 °C, and the average flow-through time of the solvent and dilute alginate samples was determined to calculate the relative (η_rel_) and specific viscosity (η_spec_). Five measurements for each sample were performed with 5 min of temperature equilibrium in between. The average times were corrected by subtracting the corresponding Hagenbach value before calculating η_rel_ and η_spec._ The relative viscosity is given by Equation (1):

(1) $$\eta_{\mathrm{rel}}=\frac{\eta }{\eta_{0}}=\frac{t}{t_{0}}$$

where t is equal to the corrected flow-through time and t_0_ is equal to the corrected flow-through time of the solvent. The solvent is the 0.1 M NaCl solution. The specific viscosity is calculated according to Equation (2):

(2) $$\eta_{\mathrm{spec}}=\frac{\left(\eta -\eta_{0}\right)}{\eta }=\eta_{\mathrm{rel}}-1.$$

To determine the intrinsic viscosity, [η], ln(η_rel_)/C, and η_spec_/C were plotted against the concentration (C in mg/mL) and extrapolated to zero concentration. The intersect of the y-axis for the two curves equals the intrinsic viscosity, which can be seen from Equations (3) and (4).

(3) $$\frac{\eta_{\mathrm{spec}}}{C}=\left[\eta \right]+k_{H}\;{\left[\eta \right]}^{2}C$$

(4) $$\frac{\ln\eta_{\mathrm{rel}}}{C}=\left[\eta \right]+k_{K}\;{\left[\eta \right]}^{2}C$$

k_H_ and k_K_ are the Huggins and Kraemer constants, respectively.

### 4.3. FTIR Measurements

Alginate solutions and calcium-alginate gels with various amounts of deuterated glucose prepared in D_2_O were studied by FTIR spectroscopy (Model Spectrum 65; PerkinElmer, Waltham, MA, USA) coupled to an ATR accessory (ZnSe crystal). FTIR spectra were averaged over 32 scans in the range of 4000–525 cm^−1^ and recorded with a resolution of 4 cm^−1^.

### 4.4. Low Field-Nuclear Magnetic Resonance (LF-NMR) Measurements

The proton relaxation studies were carried out on a Minispec mq20 NMR spectrometer (Bruker, Billerica, MA, USA) operating at 19.65 MHz. The transverse relaxation time T2 was obtained with the Carr–Purcell–Meiboom–Gill (CPMG) pulse sequence. The pulse times of 90° (P1) and 180° (P2) were 2.5 μs and 5.0 μs, respectively, and the interval between 90° and 180° pulse was 2 ms. The sampling points were 12,500, and the recycle delay time between each scan was 2 s. It must be noted that all the preparations (alginate solution and calcium-alginate gels) were made in heavy water (D_2_O) and that the glucose used was partially deuterated. Thus, the NMR signal may come from non-exchangeable protons from alginate chains and from glucose and therefore, one-exponential equation fitted satisfactorily (R^2^ ≈ 0.999) the experimental decay of the magnetization vector (Equation (5)).

I = I_0_ exp(−t/T2) (5)

where T2 and I_0_ are the proton spin–spin relaxation time and the intensity, respectively.

### 4.5. Small-Angle Neutron Scattering Experiments (SANS)

Small-angle neutron scattering (SANS) measurements were performed on the PAXY diffractometers at Laboratoire Léon Brillouin. Four configurations were used with various neutron wavelengths and sample-detector distances to cover the 0.002–0.5 Å^−1^ scattering vector range (λ = 15 Å, D = 6.7 m; λ = 8.5 Å, D = 5 m; λ = 5 Å, D = 3 m; λ = 5 Å, D = 1 m). Alginate/glucose solutions and gels were prepared with D_2_O and with deuterated glucose. This enabled (i) obtaining a good coherent contrast between the solvent and the alginate chains, (ii) matching the contribution from glucose units, and (iii) reducing incoherent scattering as much as possible.

Solutions containing 1 wt % alginate and 0, 15, 30, and 45 wt % deuterated glucose were prepared by weighting the relevant amounts of alginate and deuterated glucose that were added into to the 0.1M NaCl heavy water solution and then intensely stirring; 10 mL of each solution was prepared. The samples were left to dissolve overnight in closed vials. Each alginate/deuterated glucose solution was divided in two equal parts, one of them being used to make gels. Gels were prepared 24 h prior to the experiments by adding a CaCO_3_/GDL combination at the same concentrations as for gels used for mechanical tests. After the gelation process, 2 mm-thick cylindrical hydrogels slices were placed between two quartz windows separated by a 2 mm spacer in specific home-made cylindrical cells described in reference [30]. During measurements, the incoming neutron beam was perpendicular to the faces of the cylinder. Solutions were placed in quartz cells with a thickness of 2 mm. Standard corrections were applied for sample volume, neutron beam transmission, empty cell signal, and detector efficiency to the raw signal to obtain scattering in absolute units, and then, the signal from the pure buffer was subtracted.

The low q part of the scattered intensity was fitted to a Debye–Bueche function (Equation (6)) that is well suited for a two-phase system showing heterogeneities [34]:

(6) $$I\left(q\right)=k\frac{1}{{\left(1+q^{2}\Xi^{2}\right)}^{2}}$$

where *k* is a prefactor linked to the concentration and contrast between the two phases and *Ξ* is the characteristic size of inhomogeneities.

### 4.6. Viscosity of Glucose Solutions

The shear viscosity of the glucose solutions was measured in a strain-controlled rheometer (ARG2, TA Instrument, New Castle, DE, USA) using a 40 mm cone. Paraffin oil was added at the edge of the sample, and a solvent trap was used to prevent the sample from drying during the measurements. Measurements were performed at 25 °C. The shear viscosity was measured using a steady-state flow sweep by increasing the shear rate from 0.1 to 100 s^−1^. Measurements were done in duplicates

### 4.7. Mechanical Properties

Alginate and glucose powders were mixed and slowly added to 0.1 M NaCl solution whilst stirring until complete dilution. Gels were formed by adding 15 mM CaCO_3_ followed by 30 mM GDL under constantly mixing and quickly poured into Teflon cylindrical molds (h = 12.5 mm; d = 12.5). The molds were sealed, and the samples were allowed to set and equilibrate at room temperature for 48 h, after which they were carefully removed from the molds and immediately measured. All gels had the same alginate concentration, 1 wt %, and varying amounts of glucose: 0, 15, 30 and 45 wt %. The mechanical properties of the cylindrical gels were measured in an Instron testing machine (Instron 5542, Norwood, MA, USA) equipped with a 500 N load. Two types of experiments were performed: a compression test at rate of 0.01 mm/s until 50% strain, and a creep test by compressing the samples to 20% strain using an initial crosshead speed of 0.01 mm/s per second. The stress response upon relaxation of the gel was studied for 200 s. For each type of gel, 10 replicates were measured. True stress and true strain values were calculated by the instrument software Blue hill. The true stress at fracture, true strain at fracture, and Young’s modulus were obtained.

The compression–relaxation curves were fitted to a nonlinear poroelasticity model. In the model, a nonlinear strain-dependent factor is applied to the Young’s modulus, and the governing equations are:

(7) $$\text{Continuity}\;\text{equation}:\;\nabla \cdot \left(\sigma^{s}v^{s}+\sigma^{f}v^{f}\right)=0$$

(8) $$\text{Momentum}\;\text{equation}:\;\nabla \cdot \sigma^{\alpha }+\pi^{\alpha }=0,\;\alpha =s,f$$

(9) $$\text{constitutive}\;\text{equation}:\;\sigma^{f}=\phi^{f}pI$$

(10) $$\sigma^{s}=\phi^{s}pI+\frac{E}{3\left(1-2\upsilon \right)}e^{\gamma \epsilon }I$$

(11) $$\pi^{s}=\pi^{f}=\frac{\eta {\left(\phi^{f}\right)}^{2}}{\kappa }\left(v^{f}-v^{s}\right)$$

where σ is the stress tensor, v is the velocity vector, π is the diffusive body force vector, ϕ is the volume fraction, k is the permeability, η is the fluid viscosity, and p is the interstitial fluid pressure. The superscripts s or f denote the solid or fluid phase, respectively. The nonlinear factor $e^{\gamma \epsilon }$ is applied to the Young’s modulus E to capture the nonlinear stress–strain behavior during the compressive phase of the test, while avoiding more complex representations requiring additional fitting parameters and thus additional experiments [35]. Here, ε represents linear strain and γ is an adjustable constant. The fluid viscosity was taken as that for glucose solutions at the analyzed concentrations (1.0, 3.3 ± 0.1, 3.7 ± 0.7, and 8.5 ± 0.3 mPa s for 0 wt %, 15 wt %, 30 wt %, and 45 wt %, respectively (Figure S5)). The model was solved using the finite elements method in axisymmetric coordinates through MATLAB^®^.

### 4.8. Statistical Data Analysis

Statistical analysis of Young’s modulus and permeability data was performed using IBM SPSS Statistics Data Editor (version 28.0.1.0, Chicago, IL, USA). A post hoc Tukey’s test (*p* < 0.05) was used to determine the significant differences between mean values of independent replicates.

## Data Availability

Not applicable.

## Supplementary Materials

The following supporting information can be downloaded at https://www.mdpi.com/article/10.3390/gels8020071/s1, Figure S1: Huggins and Kraemer plots constructed for the determination of intrinsic viscosity of alginate. Huggins (η_spec_/C) [squares], and ln η _rel_/C [circles] against the concentration of alginate in 0.1 M NaCl. All measurements were performed at T = 25 °C. The error bars correspond to the standard deviation from 5 runs. Note that above a concentration of 0.2 mg/mL the errors bars are very small and not visible at the selected scale; Figure S2: FTIR spectra of alginate solution (**A**) and calcium-alginate gels (**B**) with different concentrations of deuterated glucose (0, 15, 30 and 45% (wt)). Notice that typical alginate peaks are very weak in 0% glucose due to low concentration of the sample; Figure S3: Evolution of the water activity (aw) for alginate solutions with different glucose concentrations (0, 15, 30 and 45% (wt)). Line is a guide for the eye; Figure S4: SANS scattering curves for solutions, unshifted curves. Errors bars are calculated from standard deviation; Figure S5: Shear viscosity of glucose solutions at different concentrations (15, 30 and 45 wt %). Error bars represent standard error of two replicates.
