# Supplementary material for: Impact of Glucose on the Nanostructure and Mechanical Properties of Calcium-Alginate Hydrogels
Source: Gels. 2022 Jan 22;8(2):71. doi: 10.3390/gels8020071 (PMC8871698; doi:10.3390/gels8020071)
Supplement: Supplementary file 1 [file gels-08-00071-s001.zip › gels-1521668-supplementary.pdf]

gels

Supplementary

Impact of Glucose on the Nanostructure and Mechanical Properties of Calcium-Alginate Hydrogels

Patricia Lopez-Sanchez [1,*], Ali Assifaoui [2], Fabrice Cousin [3], Josefine Moser [4], Mauricio R. Bonilla [5] and Anna Ström [1]

1 Department of Chemistry and Chemical Engineering, Chalmers University of Technology, 412 96 Gothenburg, Sweden; patlop@chalmers.se , anna.strom@chalmers.se
2 Unité Mixte de Recherche Procédés Alimentaires et Microbiologiques, Université de Bourgogne Franche-Comté (UBFC), UMR PAM A 02.102, F-21000 Dijon, France; Ali.Assifaoui@u-bourgogne.fr
3 Laboratoire Léon Brillouin, Université Paris-Saclay, UMR 12, CEA-CNRS, 91191 Gif Sur Yvette, France; fabrice.cousin@cea.fr
4 Department Bioeconomy and Health, Research Institutes of Sweden RISE, 41276 Gothenburg, Sweden; josefinemoser@gmx.de
5 Basque Center for Applied Mathematics BCAM, ,48009 Bilbao, Spain; mrincon@bcamath.org
* Correspondence: patlop@chalmers.se

Citation: Lopez-Sanchez, P.; Assifaoui, A.; Cousin, F.; Moser, J.; Bonilla, M.R. Impact of Glucose on the Nanostructure and Mechanical Properties of Calcium-Alginate Hydrogels. *Gels* **2022**, *8*, 71. https://doi.org/10.3390/gels8020071

Academic Editors: Maria Valentina Dinu

Received: 7 December 2021
Accepted: 18 January 2022
Published: 22 January 2022

Publisher's Note: MDPI stays neutral with regard to jurisdictional claims in published maps and institutional affiliations.

Copyright: © 2022 by the authors. Licensee MDPI, Basel, Switzerland. This article is an open access article distributed under the terms and con-

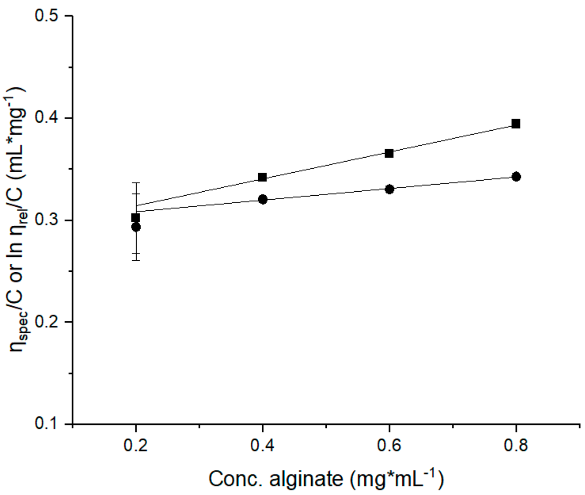

Figure S1. Huggins and Kraemer plots constructed for the determination of intrinsic viscosity of alginate. Huggins (η_{spec}/C) [squares], and ln η_{rel}/C [circles] against the concentration of alginate in 0.1 M NaCl. All measurements were performed at T = 25 °C. The error bars correspond to the standard deviation from 5 runs. Note that above a concentration of 0.2 mg/mL the errors bars are very small and not visible at the selected scale.

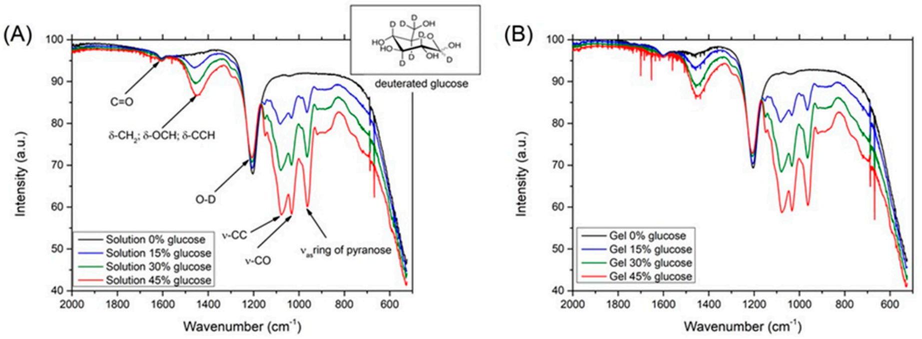

ditions of the Creative Commons Attribution (CC BY) license (https://creativecommons.org/licenses/by/4.0/).

Figure S2. FTIR spectra of alginate solution (**A**) and calcium-alginate gels (**B**) with different concentrations of deuterated glucose (0, 15, 30 and 45 wt %) Notice that typical alginate peaks are very weak in 0% glucose due to low concentration of the sample.

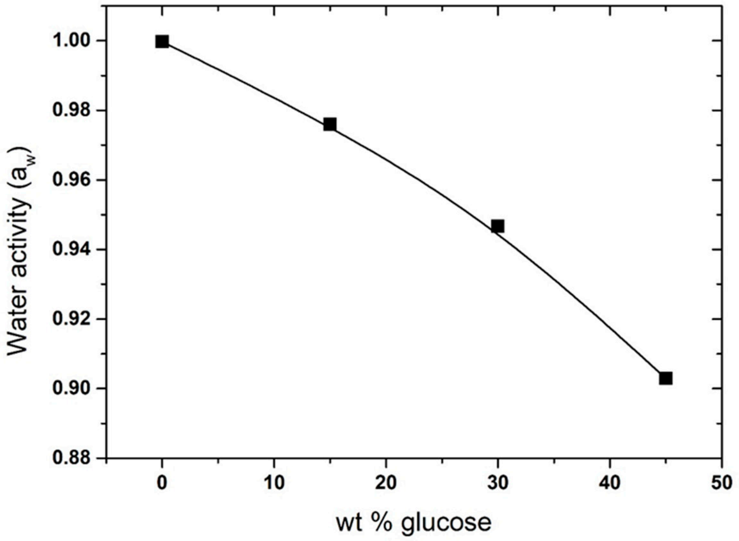

Figure S3. Evolution of the water activity (aw) for alginate solutions with different glucose concentrations (0, 15, 30 and 45 wt %). Line is a guide for the eye.

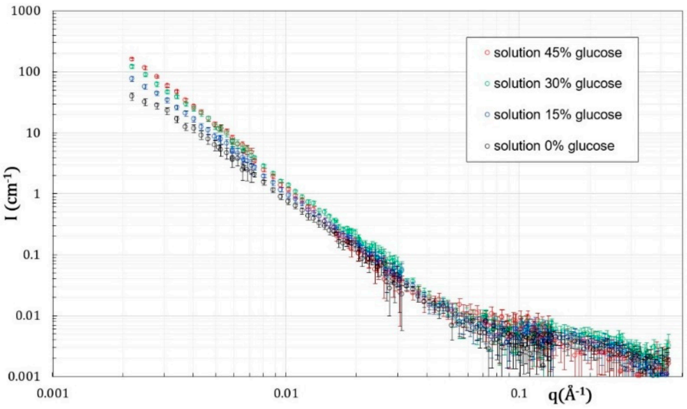

Figure S4. SANS scattering curves for alginate/deuterated glucose solutions prepared in D2O. Unshifted curves. Errors bars are calculated from standard deviation.

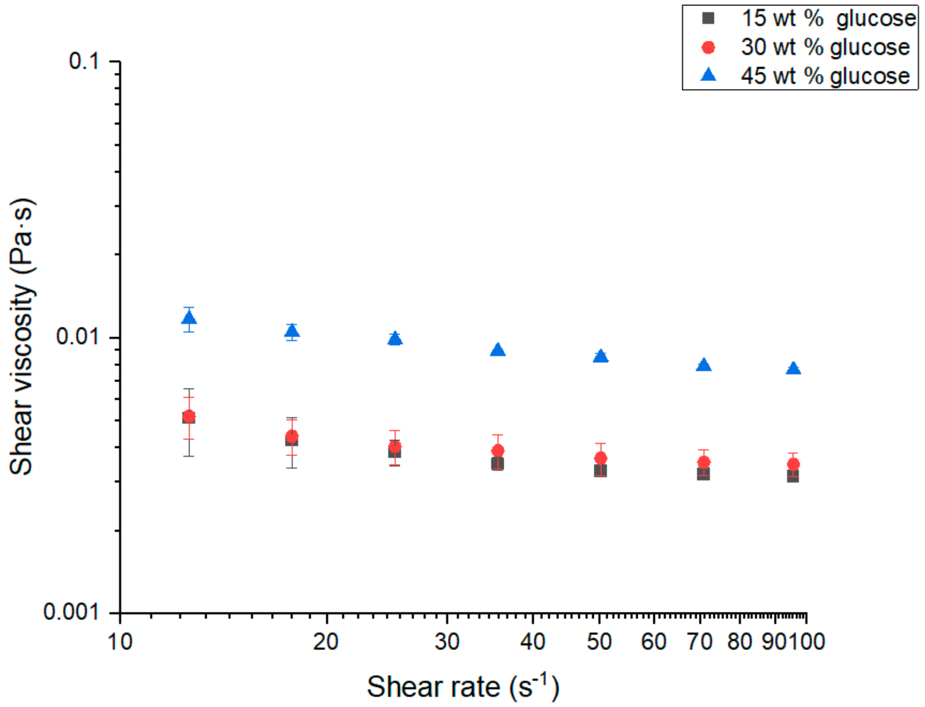

Figure S5. Shear viscosity of glucose solutions at different concentrations (15, 30 and 45 wt %). Error bars represent standard error of two replicates.
